# Cytokine-induced killer cells/dendritic cells and cytokine-induced killer cells immunotherapy for the treatment of esophageal cancer: A meta-analysis

**DOI:** 10.1097/MD.0000000000024519

**Published:** 2021-04-02

**Authors:** Xin Yuan, An Zhi Zhang, Yi Lin Ren, Xue Li Wang, Chen Hao Jiang, Lan Yang, Chun Xia Liu, Wei Hua Liang, Li Juan Pang, Wen Yi Gu, Feng Li, Jian Ming Hu

**Affiliations:** a Department of Pathology, the First Affiliated Hospital, Shihezi University School of Medicine, Xinjiang, China; b Australian Institute of Bioengineering and Nanotechnology, University of Queensland, QLD, Australia; c Department of Pathology, Beijing Chaoyang Hospital, Capital Medical University, Beijing, China.

**Keywords:** cytokine-induced killer cells, dendritic cells, esophageal cancer, immunotherapy, meta-analysis

## Abstract

**Objectives:**

This meta-analysis was designed to systematically evaluate whether autologous cytokine-induced killer cells (CIK) or dendritic cells and cytokine-induced killer cells (DC-CIK) immunotherapy combined with chemotherapy can improve the therapeutic effect and safety of chemotherapy in esophageal cancer (EC).

**Materials and methods:**

Randomized controlled trials (RCTs) were electronically searched databases including CNKI, WanFang, WeiPu, CBMDisc, PubMed, Web of Science, EMbase, the Cochrane Library, and Clinical Trials. The databases were searched for articles published until June 2019. Two researchers independently screened the literature, extracted data, and evaluated the quality of the included literature. Meta-analysis was performed using RevMan5.3.

**Results:**

Seventeen studies (1416 participants) were included. The differences between CIK/DC-CIK combination chemotherapy and chemotherapy alone were significant. The results displayed that the number of CD3^+^, CD4^+^, CD4^+^/CD8^+^, and NK cells was significantly increased after 1 to 2 weeks of treatment with CIK/DC-CIK cells in the treatment group (all *P* < .05). In addition, the results shown that 1-year overall survival was significantly prolonged (*P* < .0001) and quality of life was improved (*P* = .001) in EC chemotherapy combined with immunotherapy groups compared with conventional treatment. Furthermore, cytokine expression levels of interleukin 2 (IL-2), tumor necrosis factor α (TNF-α), and interleukin 12 (IL-12) were significantly increased (*P* = .0003) as well as the levels of immunoglobulins were elevated (*P* < .00001). Serum levels of tumor marker molecules, carcinoembryonic antigen (CEA), carbohydrate antigen (CA)-199, and CA-125 were lower in treatment groups than that of control groups (*P* < .00001). No fatal adverse reactions were noted (*P* = .04).

**Conclusions:**

It is safe and effective for patients to use chemotherapy combined with CIK/DC-CIK immunotherapy. Immunotherapy can simultaneously improve the antitumor immune response. Specifically, DC-CIK cells can increase T lymphocyte subsets, CIK cells, NK cells, and immunoglobulins in peripheral blood to enhance antitumor immunity. Therefore, combination therapy enhances the immune function and improves the therapeutic efficacy of patients with EC.

## 1 Introduction

Esophageal cancer (EC) is a malignant tumor derived from the esophageal epithelium. It is the most common digestive tract tumor and the third most common cause of cancer-related death in China.^[1]^ Clinical results show that multidisciplinary treatment including surgery, chemotherapy, and radiotherapy has been used in EC. However, the overall prognosis is poor and the 5-year global survival rate is 10% to 25%.^[2]^ Currently, surgical treatment is the main clinical treatment for EC. However, researchers found that other treatment methods such as combined chemotherapy and radiotherapy could not completely remove small tumor lesions and metastatic cells, which increased the possibility of cancer recurrence.^[3]^ Most clinically diagnosed patients experience advanced local invasion and distant metastasis, because of which surgical eradication is not feasible. Therefore, systemic chemotherapy has become a common method for the treatment of advanced EC. However, it has severe toxic side effects. Patients with EC have a poor systemic nutritional status once the cancer progresses to the advanced stage and patients find it difficult to tolerate the side effects of chemotherapy. The overall efficacy is therefore not ideal. The chemotherapy also impairs immune function and then weakens antitumor immunity of the host, leading to a tumor response, a distinct reduction in the quality of life and survival of patients, which limit its application. Despite significant advances in procedures such as surgery and chemoradiotherapy, the prognosis of patients remains poor, and new therapies are an urgent need to improve the quality of life and survival of EC patients. The key to successful treatment is the repair and rebuilding of antitumor immunity and improvement in tumor response based on chemotherapy of patients with advanced EC.

Immunodeficiency is often considered to be a critical factor in the recurrence and metastasis of EC patients. Many scientists are studying new treatments to improve efficacy against advanced EC. Recent data suggest that adoptive immunotherapy is a safe and feasible treatment for advanced tumors.^[4]^ Cytokine-induced killer (CIK) cells were first described by Schmidt-Wolf et al in 1991. The group also performed the first clinical trial involving CIK cells in cancer patients in 1999.^[5]^ In April 2010, the US Food and Drug Administration (FDA) approved the first autologous cellular immunotherapy, Sipuleucel-T, indicating that immunotherapy has enormous potential for cancer treatment.^[6]^ In recent years, cancer immunotherapy has rapidly developed, including tumor-infiltrating lymphocytes, natural killer cells (NK), dendritic cells (DCs), and CIK cells (DC-CIK cells). Among them, the combination of DCs and CIK cells is the focus of our current research. DCs, which are innate immune cells, are by far the most powerful antigen-presenting cells, capable of directly ingesting, processing, and presenting antigens and thereby stimulating the activation of primary T cells in the body.^[7]^ They are the initiators of the immune response. DCs capture and process tumor-associated antigens, and then activate antigen-specific cytotoxic T lymphocytes and induce an antitumor immune response, thus being an important regulatory role in the host immune response.^[8]^ Furthermore, the study reported that therapeutic efficacy of DCs vaccine had minimal adverse effects with no autoimmunity in hepatocellular carcinoma.^[9]^ CIK cells are cytokine-induced killer cells that mediate tumor cell apoptosis or directly cause cell death. Cytokines such as IL-2 and interferon gamma (IFN-γ) are secreted during the process. CIK cells have characteristics such as rapid proliferation, high tumoricidal activity, a broad spectrum of tumor killing, and strong recognition ability.^[10]^

With the development of cellular immunology and molecular biology, biological immunotherapy has been gradually applied in the treatment of malignant tumors. DCs combined with CIK cells in biological immunotherapy can effectively improve the autoimmunity of cancer patients while treating malignant tumors, thus further improving the survival rate and time for these patients.^[11]^ Therefore, CIK cells or coculture of DC cells with CIK cells form the CIK/DC-CIK therapy, which has become an important cellular immunotherapy. CIK/DC-CIK cell therapy has been extensively studied and the combination of CIK/DC-CIK cell therapy with chemotherapy has been used in treating malignancy.^[12]^ In clinical practice, treatment with different combinations of CIK/DC-CIK cells and chemotherapy has outstanding diversity. These may have different effects on clinical outcomes and may, therefore, be an important factor hindering the success of individualized combination therapies. Then, whether CIK cells/DC-CIK cells treatment can repair and rebuilding antitumor immunity is the primary issue governing successful treatment. Antitumor immunity is indicated by indicators such as T lymphocyte subsets and NK cells, which are of great value for early judgment of the clinical efficacy of treatment with CIK/DC-CIK cells. Previous studies reported that DC-CIK cells therapy significantly increased the cells proportion of CD3^+^, CD4^+^, and the ratio of CD4^+^/CD8^+^ cells.^[13,14]^ Furthermore, these improved tumor response in EC. However, due to sample size variability and the use of non-standardized clinical trial protocols, the exact therapeutic effect is not yet clear. Whether CIK cells/ DC-CIK cells treatment can increase the number of CD8+ T cells remains controversial. Whether combined chemotherapy can enhance antitumor immunity, prolong patient survival, and improve tumor response remains unclear. To address these issues, we conducted extensive searches and a rigorous data analysis. To date, some studies on immune combined chemotherapy for other tumors have been published,^[15–18]^ this meta-analysis includes CIK cells and DC-CIK cells treatment. Therefore, the purpose of this study was to explore whether CIK cells/DC-CIK cells immunotherapy was efficacious and reduced adverse reactions. We also investigated whether the treatment could repair and rebuild patients’ antitumor immunity after chemotherapy, so as to determine the best combination with chemotherapy. To provide the best basis for the effectiveness and safety of treatment and for individualized combination therapy, we systematically reassessed all relevant studies.

## 2 Materials and methods

### 2.1 Search strategy

The authors independently searched all published studies in Chinese Biomedical Literature (CBM), the China Science Journal Full-text Database (CNKI), the China National Knowledge Infrastructure Database (VIP), Wanfang, PubMed, Web of Science (ISI), Embase, MEDLINE, and Cochrane Controlled Trials Center (CENTRAL) databases, and further searched the World Health Organization (WHO) International Clinical Trial Registration Platform (WHO-ICTRP), China Clinical Trial Registry (Chi-CTR), and US Clinical Trial (established in June 2017). Search strategy was a combination of subject terms and free words. The search terms were “esophageal cancer,” “dendritic cells,” “vaccine,” “CIK,” “DC-CIK,” “immunotherapy,” “chemotherapy,” “biotherapy,” and “clinical trials.” No language restrictions were applied. The initial search was conducted in January 2019 and updated in June 2019. Finally, we identified and evaluated all relevant systematic reviews (SR) or meta-analyses, and then selected all studies that met the inclusion criteria from the references.

### 2.2 Inclusion and exclusion criteria

The following researches were included: studies with a diagnosis of EC based on histopathology, cytological diagnostic criteria, and the tumor node metastasis (TNM) staging system were included for analysis^[19–21]^; studies in which no patient showed severe liver or kidney dysfunction; studies in which all patients only underwent surgery, radiotherapy, treatment with Chinese medicine, CIK cells treatment alone, monoclonal antibody treatment, or other cell therapies prior to enrollment. The studies we selected were randomized controlled trial (RCTs) in patients with EC. CIK cells/DC-CIK cells treatment combined with conventional chemotherapy was defined as the combination treatment group and a separate conventional chemotherapy regimen was defined as the control group. There was no limit with regarding to study type for inclusion. The following researches were excluded: patents, abstracts, general comments without specific data; repeat studies; in vitro or animal studies; research on nursing or other cancer; non-randomized controlled studies; irrelevant SR or meta-analysis; studies of cells or DC-CIK cells; studies on CIK cells/DC-CIK cells treatment combined with radiotherapy, Chinese herbal medicine, targeted therapy, and other cells therapy; CIK/DC-CIK was divided into 2 separate groups of studies.

### 2.3 Study selection

Data extraction was performed independently by 2 authors (AZZ and LYR) by using standardized methods. The literature was independently searched and collected from databases according to our inclusion criteria, and data were drawn from all selected articles. Differences in opinion were settled by discussions with the fourth author (XLW). The information collected includes the first author's name, journal, publication year, number of subjects, patient age, sex, protocol, and tumor stage. The number of patients evaluable for 1-year survival and the number of evaluable patients included information related to the study design (whether the trial reported randomization patterns, allocation concealment, and blinding); in vitro cell culture conditions and dosage of immune cells.

### 2.4 Outcome measures

We used T lymphocyte subsets and NK cells in peripheral blood to assess antitumor immunity. T lymphocyte subsets were measured based on the ratio of CD3^+^ cells, CD4^+^ T cells, CD8^+^ T cells, CIK cells (CD3^+^CD56^+^ cells), Treg cells (CD25^+^CD4^+^ cells), and CD4^+^/CD8^+^ cells. All indicators were tested after treatment by using flow cytometry (FCM) or other methods. Tumor response was measured based on objective response rate (ORR) and disease control rate (DCR) according to the WHO Solid Tumor Response Guidelines^[22]^ or Solid Tumor Response Assessment Criteria (RECIST).^[23]^ The specific indicators studied were complete response (CR), partial response (PR), stable disease (SD), progressive disease (PD), ORR equals CR plus PR, and DCR equals CR plus PR and SD.

### 2.5 Data extraction

Two authors (CHJ and LY) independently extracted all data according to a pre-designed data extraction form, including the publication time and first author, demographic characteristics, sample sizes of the experimental and control groups, CIK cell/DC-CIK cell type and usage, and their combination with chemotherapy and time. The primary outcome was antitumor immunity and the secondary outcome was tumor response.

### 2.6 Quality assessment

The quality of studies was assessed via using the Cochrane Handbook: random sequence generation, allocation concealment, blinding of participants and personnel, blinding of outcome assessment, incomplete outcome data, selective reporting, and other bias. Publication bias was generated by using a funnel plot to examine whether there was a bias towards studies.

### 2.7 Statistical analysis

Two authors (XY and AZZ) used the Review Manager Version 5.3 provided by Cochrane Collaboration to analyze the data. Relative risk, standardized mean difference, and 95% confidence interval (CI) was used to describe the 2 categorical variables and the continuous variables, respectively. Heterogeneity analysis was performed using of the chi-squared test and *I*^2^ statistic. If *P* > .1, *I*^2^ < 50%, homogeneity among multiple studies was assessed using the fixed effect model; otherwise, for data showing heterogeneity (*P* < .1, *I*^2^ > 50%), assessments were performed by a random-effects model, and when there were >8 included trials, a funnel plot was used for publication bias analysis. To further revealing the effect of CIK/DC-CIK combined with chemotherapy, a subgroup analysis was carried out to show clinical heterogeneity and its effects on T lymphocyte subsets and tumor response.

## 3 Results

### 3.1 Search results

**Figure 1:**
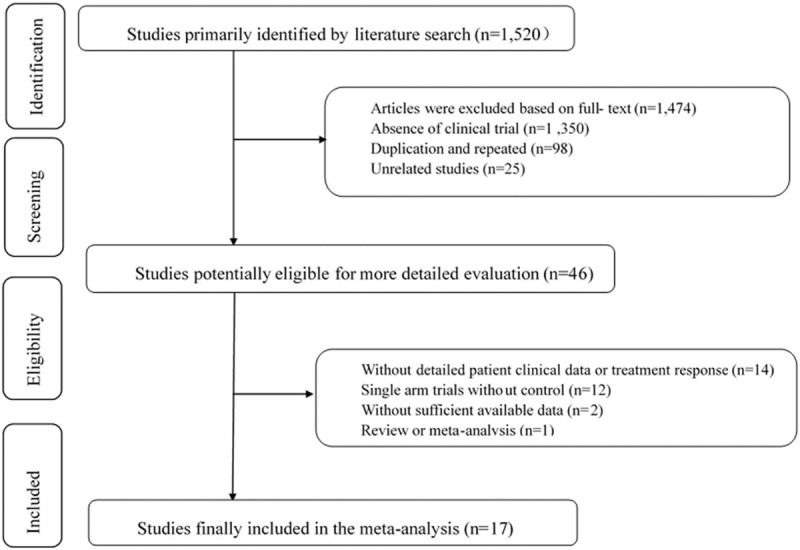
Articles retrieved and assessed for eligibility.

A total of 1520 articles were found in the initial search. After careful review of the title and abstract, 1474 articles were excluded. A total of 46 studies were selected for potential relevance. After referring to the full text, 29 articles were excluded (because 14 did not include detailed data for patient clinical characteristics or treatment response, 12 were allotted to the control group, 2 had insufficient data, and 1 was meta-analysis). Finally, 17 trials that included 1416 patients met the criteria for inclusion in this meta-analysis. The reasons for exclusion were indicated in Fig. 1.

### 3.2 Characteristics of the included studies

A total of 17 eligible trials were included, including 1416 EC patients, ranging in age from 42 to 74 years. Among these, 717 patients received CIK/DC-CIK combined with conventional therapy (combination therapy), while 699 patients received only conventional treatment. Both DC and CIK cells used in the 17 trials were taken from autologous peripheral blood. In 10 trials,^[24–33]^ DC-CIK cells immunotherapy was applied, while in the other 7 trials,^[34–40]^ only CIK cells were used. CIK/DC-CIK cells were mainly returned by intravenous infusion after chemotherapy. The number of cells per transfusion was 1–10 × 10^9^ immune cells, 2 to 6 times/cycle. The control group received chemotherapy alone, such as vinorelbine and cisplatin (NP), paclitaxel and cisplatin (TP), docetaxel and cisplatin (DP), and gemcitabine and cisplatin (GP). In most studies, patients received >1 × 10^9^ immune cells, but Qu et al^[35]^ did not provide accurate cell numbers. There were no significant differences in the clinical data between the treatment group and the control group (*P* > .05). The detailed clinical information of the trials is shown in Table 1.

**Table 1:** Characteristics of included studies.

Study (y)	Tumor stage	Patients, exp/cont groups	Age (mean ± SD or median) (treatment/con)		Sex (male/female)	Exp regimens	Immunotherapy method
Chang et al 2013	III–IV	33/33	66 (median)	66 (median)	43/23	CT, CIK	1 × 10^9^ CIK cells
Hu et al 2012	III–IV	37/25	ND	ND	34/28	CM, CT, CIK	>1 × 10^9^ CIK cell
Liu et al 2011	III–IV	20/20	62 (median)	62 (median)	23/17	CT, CIK	0.6–1.6 × 10^10^ CIK cells
Qu et al 2015	IV	100/100	56.3 ± 7.5 (mean)	56.3 ± 7.5 (mean)	ND	CT, CIK	ND
Shu et al 2015	II–III	30/30	57 (median)	59 (median)	39/21	RT, CT, CIK	5 × 10^10^ CIK cells
Wang et al 2014	I–IV	62/62	ND	ND	92/32	CT, DC-CIK	2 × 10^10^ DC-CIK cells
Wen et al 2015	I–II	41/35	59 (median)	59 (median)	43/33	RT, CT, DC-CIK	ND
Xi et al 2015	II–IIIB	26/26	60 (median)	62 (median)	30/22	Surgery, CT, DC-CIK	3–4 × 10^9^ DC-CIK cells
Xu et al 2018	IV	28/28	58 (median)	56 (median)	36/20	CT, DC-CIK	ND
Xu et al 2010	III–IV	21/25	45 (median)	42 (median)	27/19	CT, CIK	>5 × 10^9^ CIK cells
Yan et al 2015	I–IV	34/34	70.5 ± 2.9 (mean)	71.6 ± 2.2 (mean)	45/23	RT, DC-CIK	5 × 10^9^ CIK cells 5 × 10^7^ DC cells
Yang et al 2016	ND	35/35	64.9 ± 1 (mean)	65.3 ± 1.3 (mean)	35/35	CT, DC-CIK	1.5 × 10^10^ DC-CIK cells
Yang et al 2015	ND	100/100	70.2 ± 7.3 (mean)	72.3 ± 6.9 (mean)	147/53	CT, DC-CIK	ND
Zhang et al 2017	II–IIIB	30/30	64 ± 5.7 (mean)	64.2 ± 5.9 (mean)	35/25	Surgery, CT, DC-CIK	3–4 × 10^9^ DC-CIK cells
Zhang et al 2016	IV	32/28	56.9 ± 8.9 (mean)	56 ± 10.4 (mean)	37/23	CT, DC-CIK	>6 × 10^9^ DC-CIK cells
Zhao et al 2015	IIIB–IV	50/50	55.9 ± 6.3 (mean)	56.5 ± 5.5 (mean)	62/38	CT, DC-CIK	ND
Zhu et al 2014	ND	38/38	59.6 ± 1.3 (mean)	59.8 ± 1.4 (mean)	43/33	CT, CIK	ND

The table summarizes patients’ basic information regarding the tumor stage, treatment, regimens, cases, age and cell doses of the immunotherapy. CIK = cytokine-induced killer cell, CM = Chinese medicine (Huisheng oral liquid), Cont = control group, CT = chemotherapy, DC = dendritic cell, Exp = experimental group, ND = not determined, RT = radiotherapy.

### 3.3 Methodological bias risk

**Figure 2:**
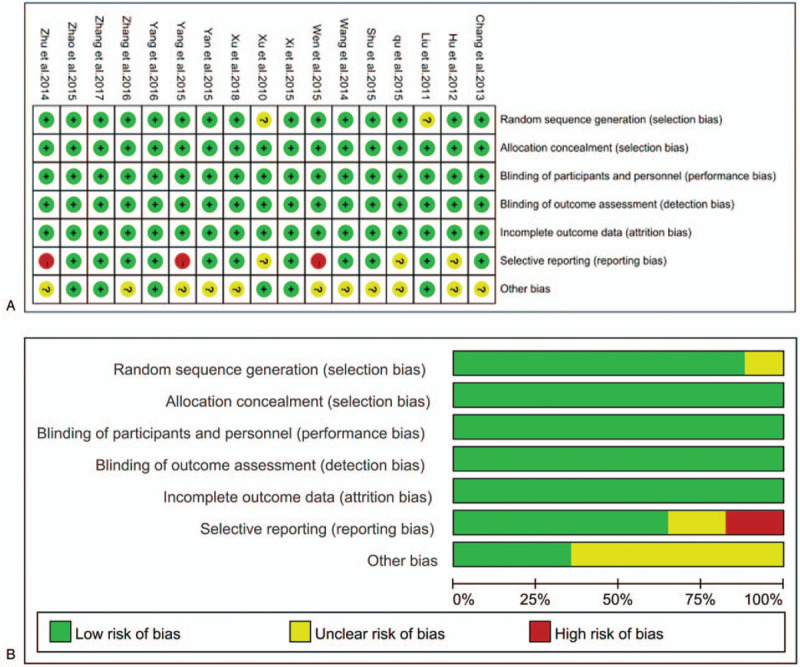
A. Risk of bias summary: a review of authors’ judgments for each risk of bias item for included studies. B. Risk of bias graph: a review of authors’ judgments for each risk of bias item presented as percentages across all included studies.

Although there are many approaches to randomization that are known to effectively conceal the randomization sequence, the use of sequentially numbered, opaque sealed envelopes (SNOSE) is both cheap and effective. This research applies the SNOSE process to achieve allocation concealment. Among the 17 trials, 15 trials used randomization, while the other 2 trials did not provide detailed information on allocation concealment. No trial provided detailed information on blinding. The risk assessment for bias is demonstrated in Fig. 2.

### 3.4 T lymphocytes and subsets

**Figure 3:**
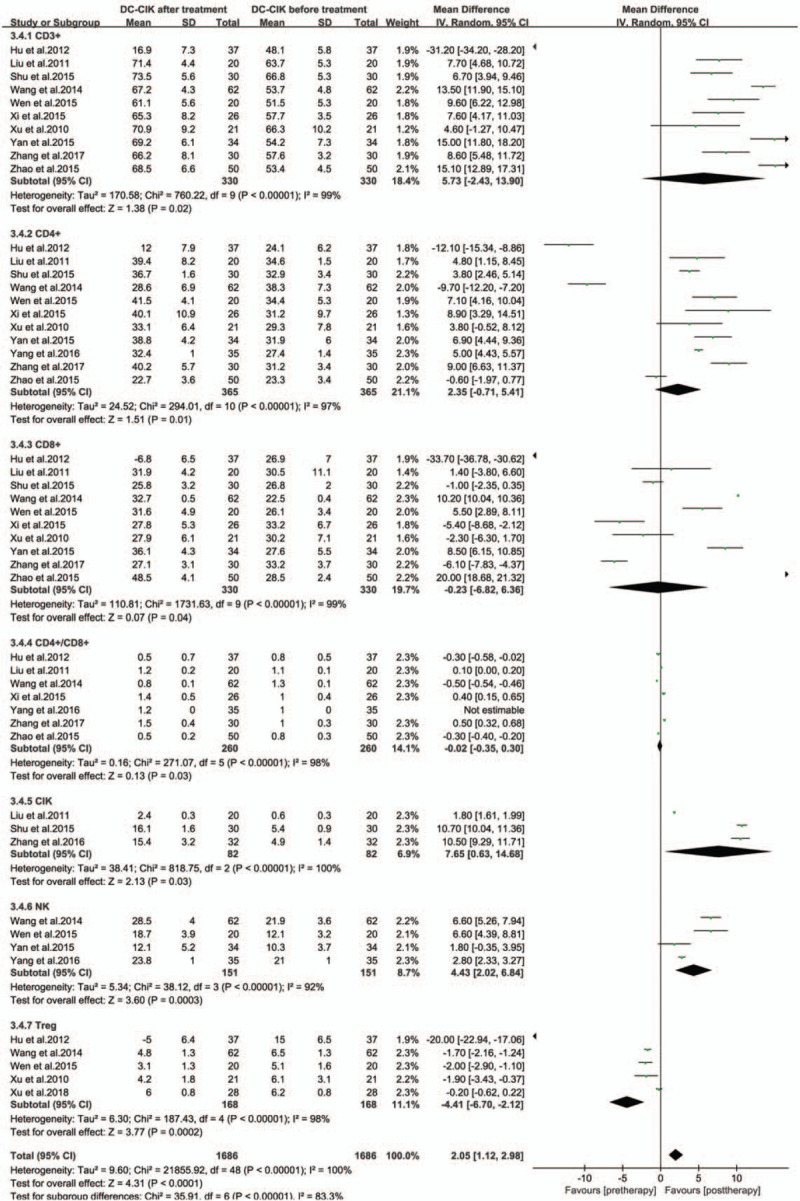
Forest plot of immunophenotype assessment before and after treatment with CIK/DC-CIK. The random-effects meta-analysis model was used. CI = confidence interval, CIK/DC-CIK = immunotherapy with cytokine-induced killer cells or a combination of dendritic cells and cytokine-induced killer cells.

Immune function was assessed by comparing changes in T lymphocyte subsets and NK cells before and after treatment in different studies. Overall, there is increase in CD3+ cells (MD; 5.73 [–2.43, 13.90]), CD4+ (MD; 2.35 [–0.71, 5.41]), CIK (MD; 7.65 [0.63, 14.68]), and NK (MD; 4.43 [2.02, 6.84]). However, CD4+/CD8+ (MD; –0.02 [–0.35, 0.30]) and Treg (MD; –4.41 [–6.70, –2.12]) were decreased after 1 to 2 weeks of treatment with CIK/DC-CIK cells in the treatment group^[34]^ (Fig. 3, all *P* < .05).

### 3.5 Tumor responses

**Figure 4:**
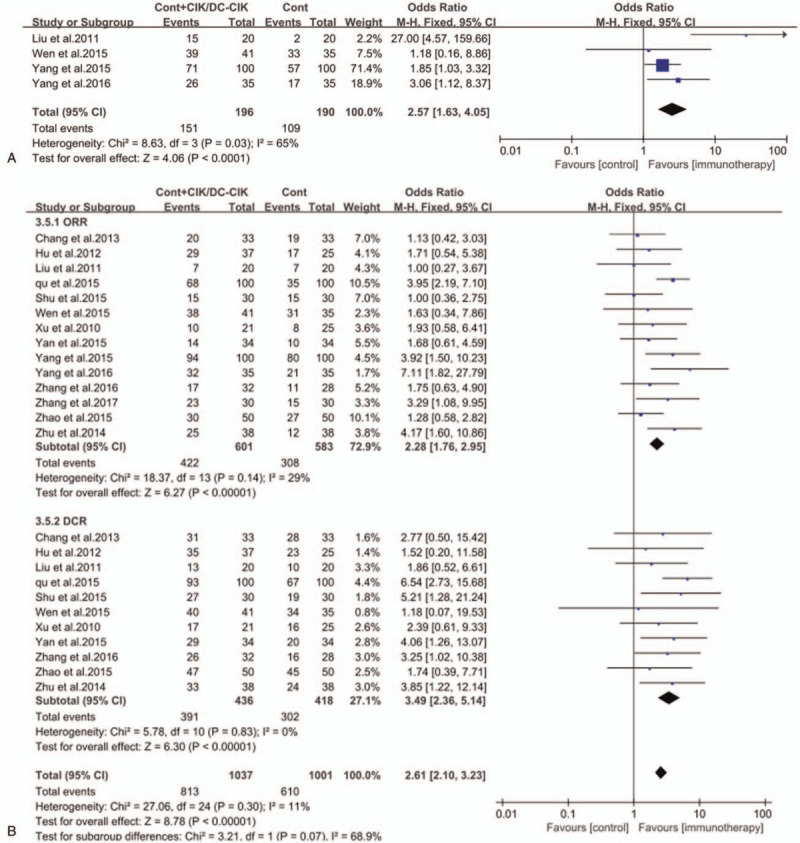
Forest plots of the comparisons of (A) overall survival (OS) and (B) overall response rate (ORR) and disease control rate (DCR). The fixed-effects meta-analysis model (Mantel–Haenszel method) was used. CI = confidence interval, CIK/DC-CIK = immunotherapy with cytokine-induced killer cells or combination of dendritic cells and cytokine-induced killer cells; cont = conventional therapy, M–H = Mantel–Haenszel method.

**Figure 5:**
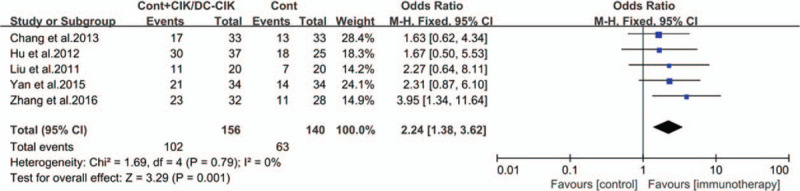
Forest plots of the comparisons of quality-of-life improved rate (QIR). The fixed-effects meta-analysis model (Mantel–Haenszel method) was used.

The tumor response was assessed by comparing objective response rate (ORR) and disease control rate (DCR) before and after treatment in different studies. Compared with patients treated with chemotherapy alone, it was significantly prolonged overall survival time (OR; 2.57 [1.63, 4.05]) and improved ORR (OR; 2.28 [1.76, 2.95]), DCR (OR; 3.49 [2.36, 5.14]) (Fig. 4, all *P* < .0001) and quality-of-life improved rate (QIR) (OR; 2.24 [1.38–3.62]) (Fig. 5, *P* = .001) in CIK/DC-CIK cells immunotherapy and chemotherapy combination therapy groups.

### 3.6 Serum immunoglobulin content

**Figure 6:**
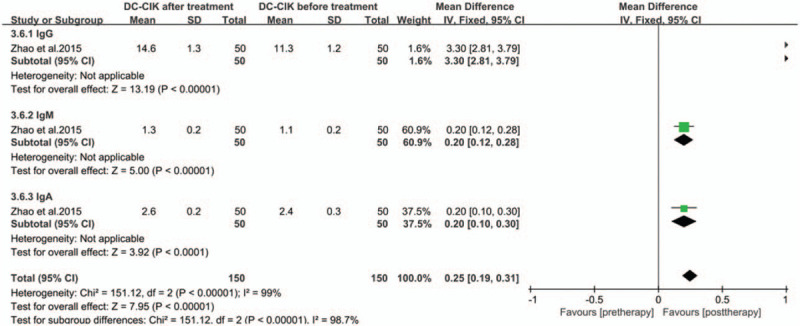
Forest plots of the comparisons of immunoglobulin levels in serum.

**Figure 7:**
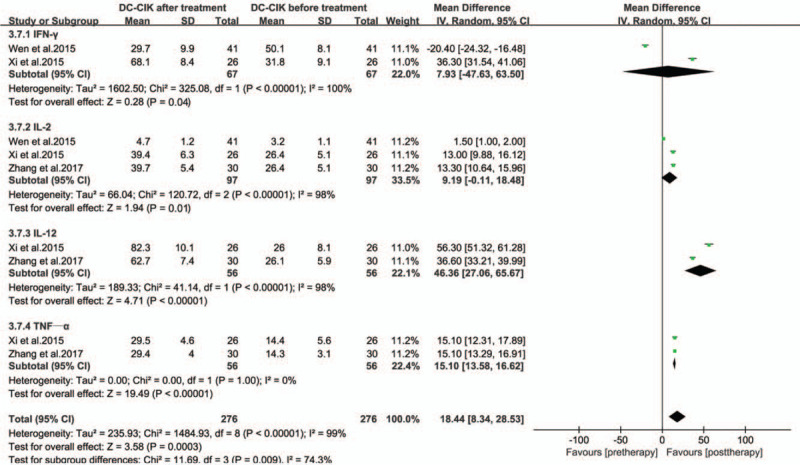
Forest plots of the comparisons of cytokine levels.

**Figure 8:**
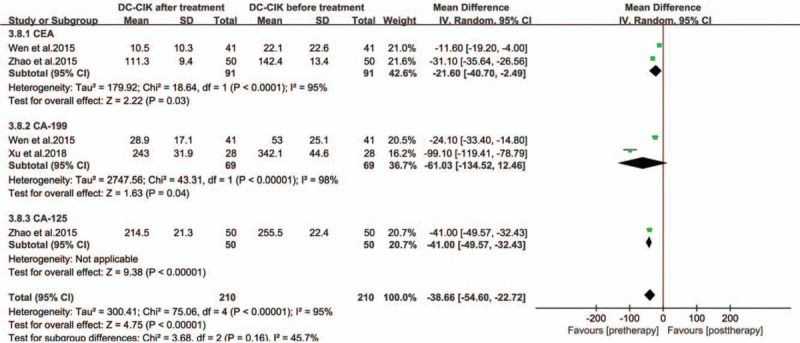
Forest plots of the comparisons of tumor marker levels in serum.

The levels of peripheral serum immunoglobulins IgG, IgM, and IgA in the treatment group were higher than those before treatment (Fig. 6, all *P* < .00001). The production of cytokines IFN-γ, IL-2, IL-12, and TNF-α shown a significant increase in the treatment groups (Fig. 7, all *P* < .05). The levels of peripheral serum carcinoembryonic antigen (CEA) and carbohydrate antigen (CA-199 and CA-125) were obviously lower in immunotherapy patients than those before treatment (Fig. 8, all *P* < .05).

### 3.7 Toxicity and side effects

**Figure 9:**
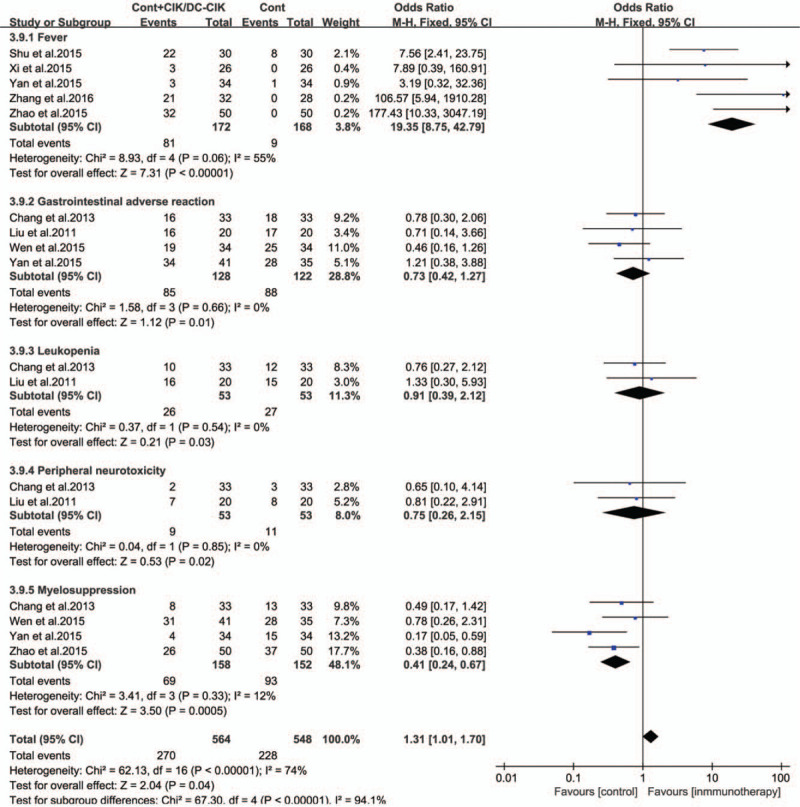
Forest plots of the comparisons of side effects.

Adverse reactions observed during the course of treatment are listed in Table 2. The most common adverse reaction was fever. Chills, rashes, and headaches have also been reported, but they are not common. There were no cases of severe diarrhea, shock, abnormal blood test results, liver dysfunction, or renal insufficiency. In addition, the incidence of myelosuppression and gastrointestinal adverse reactions in the immunotherapy group was significantly lower than that in the control group (Fig. 9, all *P* < .05).

**Table 2:** Side effects of CIK/DC-CIK treatment and myelosuppression.

					Gastrointestinal adverse reaction		Myelosuppression	
Study and year	Fever	Chills	Rash	Headache	Chemo/CIT	Control	Chemo/CIT	Control
Chang et al 2013	–	–	–	–	48.5%	54.5%	24.2%	39.4%
Shu et al 2015	74.6%	–	–	Mild	–	–	–	–
Wen et al 2015	–	–	–	–	82.9%	80%	78%	80%
Xi et al 2015	11.5%	–	–	–	–	–	–	–
Yan et al 2015	8.8%	Mild	–	–	55.9%	73.5%	11.8%	44.1%
Zhao et al 2015	64%	20%	10%	–	62%	88%	52%	74%
Zhang et al 2016	65.6%	3.1%	–	–	Mild	Mild	1–2 degree	1–2 degree

CIK = cytokine-induced killer cell, CIT = chemotherapy-induced thrombocytopenia, DC/CIK = dendritic cell–cytokine-induced killer cell.

### 3.8 Publication bias

**Figure 10:**
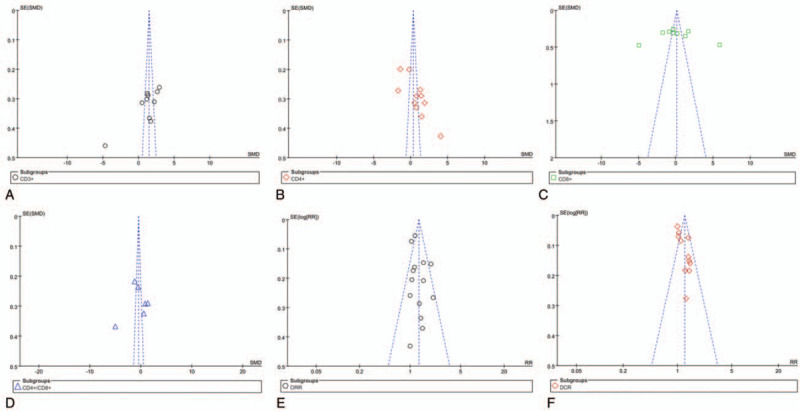
Analysis of publication bias.

To determine whether the included studies have publication bias, we present the data in the form of a funnel plot. As shown in Fig. 10, the results of poor symmetry in the meta-analysis were analyzed and evaluated by the literature elimination method, and there is no change before and after data processing.

## 4 Discussion and conclusion

With the growing body of research on malignant tumor immunotherapy, an increasing number of clinical trials have used CIK/DC-CIK cells for the treatment of malignant tumors, especially in patients with hematological tumors and solid tumors.^[39–41]^ In fact, chemotherapy can enhance the antitumor response by increasing the release of tumor antigens. It also enhances antitumor T cell function by eliminating the activity of Treg cells and myeloid-derived suppressor cells, thereby generating a more potent antitumor immune response. Therefore, it is the primary treatment for advanced EC. However, chemotherapy can only kill a certain number of tumor cells and malignant tumor cells may survive after traditional chemotherapy. Moreover, this treatment is thought to weaken the immune system and may cause various side effects that inhibit the immune function of the patients. Importantly, elimination of tiny residual lesions requires mobilization and enhancement antitumor immunity. In contrast, chemotherapy combined with immunotherapy can kill tumor cells and eliminate tiny residual lesions while strengthening the antitumor immunity weakened by chemotherapy. It is expected to become a new treatment strategy for patients with advanced cancer. Studies have shown that DC plays a crucial role in activating CIK and the secretion of cytokines such as IL-12, IFN-γ, and TNF-α.^[42]^ Coculture of DC and CIK can increase levels of cytokines (IL-2, IFN-γ, TNF-α, and IL-12) and decrease the negative regulators such as TGF-β and IL-10. After DC and CIK coculture, the proportion of CD3+ CD56^+^ cells (the main effector cells that enhance CIK cytotoxicity) increased, while the proportion of CD4^+^CD25^+^ regulatory T cells (which inhibited the anti-tumor activity of CIK) decreased. In addition, CIK cells promote DC maturation and the expression of costimulatory molecules such as CD40, CD80, and CD86. Therefore, the combination of DC and CIK lead to a significant increase in cytotoxic activity.^[43]^ DC is commonly used in active antitumor immunotherapy as a key cell for antitumor immunity. Furthermore, CIK cells exhibit strong cytotoxicity against tumor cells and freshly isolated tumor samples, including liver cancer, gastric cancer, lung cancer, and glioma. Compared with other immune cells, CIK cells show a higher proliferation rate and stronger antitumor activity in a wider range of tumors. DC and CIK cells are the most promising immune cells in the field. Therefore, the combination of chemotherapy and CIK/DC-CIK immunotherapy for EC patients has attracted widespread attention.

It has been reported that the time and dose effects of tumor chemotherapy drugs can be used in tumor therapy to break immunosuppression or tolerance and stimulate the immune response. Therefore, the therapeutic effect of CIK/DC-CIK combined with chemotherapy may be synergistic and complementary.^[44,45]^ In recent years, with the rapid development of modern molecular biology, oncology and tumor immunology, biological treatment of tumors has become the fourth new treatment mode in addition to surgery, radiotherapy, and chemotherapy. There are molecular target drugs, monoclonal antibodies, cytokines, gene therapy, and cellular immunotherapy in current tumor biotherapeutic techniques.^[46]^ Among these, adoptive immunotherapy is a potential method for treating various solid tumors, especially malignances that are hard to cure by conventional therapy. The advantage of adoptive immunotherapy is that it has the ability to stimulate and restore immunity, which can recognize and induce tumor cell death.^[47]^ CIK/DC-CIK biotherapy is a new generation of adoptive T cell immunotherapy, which is currently the most widely used form of immunotherapy at home and abroad.^[48]^ Some recent clinical trials have used CIK/DC-CIK immunotherapy combined with chemotherapy for EC. This meta-analysis examined 17 clinical trials that included 717 EC patients receiving CIK/DC-CIK immunotherapy combined with chemotherapy. Studies have shown that the incidence of fever in patients treated with autologous CIK/DC-CIK cells was as high as 44.9%, while the fever rate during conventional treatment was close to 0, indicating that the cellular immune response and the application of interferon may cause the patients show a fever reaction, which is predictable and easy to handle. The researches^[49–52]^ suggest that patients with autologous CIK/DC-CIK cell immunotherapy show fewer adverse reactions, with only a small number of patients showing fatigue, muscle soreness, headache, and other similar cold-like symptoms, which consistent with the results of our meta-analysis.

This study confirmed that combination therapy can provide better clinical efficacy and fewer adverse reactions compared with chemotherapy alone. The levels of CD3^+^, CD4^+^, CD4^+^/CD8^+^, and NK cells associated with immune function in the peripheral blood of patients were significantly higher than those before treatment, suggesting that the cellular immune function was obviously enhanced of EC patients. These findings also confirmed the safety of CIK/DC-CIK immunotherapy for EC patients and showed that all patients can tolerate the adverse effects of this treatment. In comparison with the chemoradiotherapy group, the incidence of hematological toxicity, hepatorenal toxicity, and other similar conditions did not significantly change after treatment with autologous CIK/DC-CIK cells. Only a few patients developed fever, mild headache, fatigue, and joint pain, etc. Symptoms such as soreness may resolve gradually or can be relieved by symptomatic treatment, which proves that autologous CIK/DC-CIK cell immunotherapy is safe and reliable without causing serious adverse reactions or death after treatment. Moreover, CIK/DC-CIK immunotherapy can significantly reduce the adverse events caused by chemotherapy, including leukopenia, peripheral neuritis, gastrointestinal side effects, and myelosuppression. The efficiency of EC chemotherapy was increased and the OS, ORR, DCR, and QIR were improved in combination therapy group.

However, this study had some limitations. First, there were 2 trials that did not report random assignments. All trials did not provide detailed information on blinding. Next, research on evaluation of Treg cells and cytokines needs to further expand the sample size (such as IFN-γ response has the opposite result) in this meta-analysis. Third, the number of cells also shown clinical heterogeneity, so we did not perform a subgroup analysis to reveal the effect of cell number on T lymphocyte subsets due to most studies have not reported. These limitations may have resulted in an inadequate assessment of the antitumor immunity and tumor response. Importantly, this meta-analysis is more comprehensive than previous studies on EC, covering all stages of EC, and provided a more detailed analysis of immunoglobulin changes in humoral immunity, not just T cell subsets. This study also analyzed cellular immunity to determine its diagnostic and prognostic correlation with serum tumor marker molecules. The increase in serum tumor marker molecules indicates that the immunity is reduced and the prognosis of patients is poor.

In conclusion, this study shows that the combination of chemotherapy and immunotherapy can improve the antitumor immunity, significantly improve quality of life, and effectively control tumor progression of EC patients. Moreover, no obvious toxic and side effects during combination therapy. Therefore, it is worthy of promotion and clinical application.

## Author contributions

**Conceptualization:** Wen Yi Gu.

**Data curation:** Xin Yuan, An Zhi Zhang, Xue Li Wang, Chen Hao Jiang.

**Funding acquisition:** Feng Li.

**Investigation:** Xue Li Wang, Wei Hua Liang.

**Methodology:** Yi Lin Ren.

**Resources:** Jian Ming Hu.

**Validation:** Chen Hao Jiang, Chun Xia Liu.

**Visualization:** Li Juan Pang.

**Writing – original draft:** Xin Yuan.

**Writing – review & editing:** Lan Yang.

## Correction

When originally published, affiliation a was incorrect and has been removed. Author affiliations have also been adjusted in the byline. Affiliation b was removed from all authors except for Chun Xia Liu and added to Wen Yi Gu. Affiliation c was added to Feng Li.
